# Monitoring silica core@shell nanoparticle‐bacterial film interactions using the multi‐parametric surface plasmon resonance technique

**DOI:** 10.1002/SMMD.20230012

**Published:** 2023-06-26

**Authors:** Rawand A. Mustafa, Petteri Parkkila, Jessica M. Rosenholm, Hongbo Zhang, Tapani Viitala

**Affiliations:** ^1^ Pharmaceutical Sciences Laboratory Faculty of Science and Engineering Åbo Akademi University Turku Finland; ^2^ Division of Nano and Biophysics Department of Physics Chalmers University of Technology Gothenburg Sweden; ^3^ Drug Research Program Division of Pharmaceutical Chemistry and Technology Faculty of Pharmacy University of Helsinki Helsinki Finland; ^4^ Turku Bioscience Centre University of Turku and Åbo Akademi University Turku Finland

**Keywords:** antibacterial, biofilm, cerium oxide (CeO_2_), mesoporous silica, multi‐parametric surface plasmon resonance (MP‐SPR), nanoparticle‐biofilm interactions, nanoparticles

## Abstract

In a healthcare setting, biofilms are a major source of infection and difficult to eradicate once formed. Nanoparticles (NPs) can be designed to effectively penetrate biofilms to more efficiently either deliver antibiotic drugs throughout the biofilm matrix or elicit inherent antibiofilm activity. Antibacterial cerium oxide (CeO_2_) NPs were employed as core material and coated with a mesoporous silica shell (MSN) to generate cerium oxide coated mesoporous silica NPs (CeO_2_@MSN). Detailed studies of NP‐biofilm interactions are required to rationally develop NP platforms to prevent biofilm‐related infections. This work developed and implemented a unique label‐free analysis platform for the real‐time monitoring of bacterial biofilm formation and then assessed the interactions of antibacterial NPs. An analysis platform which allows bacterial biofilms to grow and develop in situ in flow within the multi‐parametric surface plasmon resonance (MP‐SPR) instrument was established. This enabled simultaneous monitoring and detection of biofilm growth phases, structure, and interactions between differentially charged CeO_2_@MSNs and bacterial biofilms. Positively charged antibacterial NPs (polyethyleneimine functionalized CeO_2_@MSNs) were found to be the most efficient to penetrate the biofilm. The MP‐SPR analysis platform was shown to be a powerful tool for monitoring biofilm development in real‐time and to analyze biofilm properties and NP‐biofilm interactions.

## Graphical abstract

Nanoparticles (NPs) can be designed to effectively penetrate biofilms to deliver antibiotic drugs more efficiently. Antibacterial cerium oxide NPs coated with a mesoporous silica shell were developed and their interactions with biofilms were studied with a unique label‐free analysis platform developed for real‐time monitoring of bacterial biofilm formation and subsequent assessment of NP‐biofilm interactions.

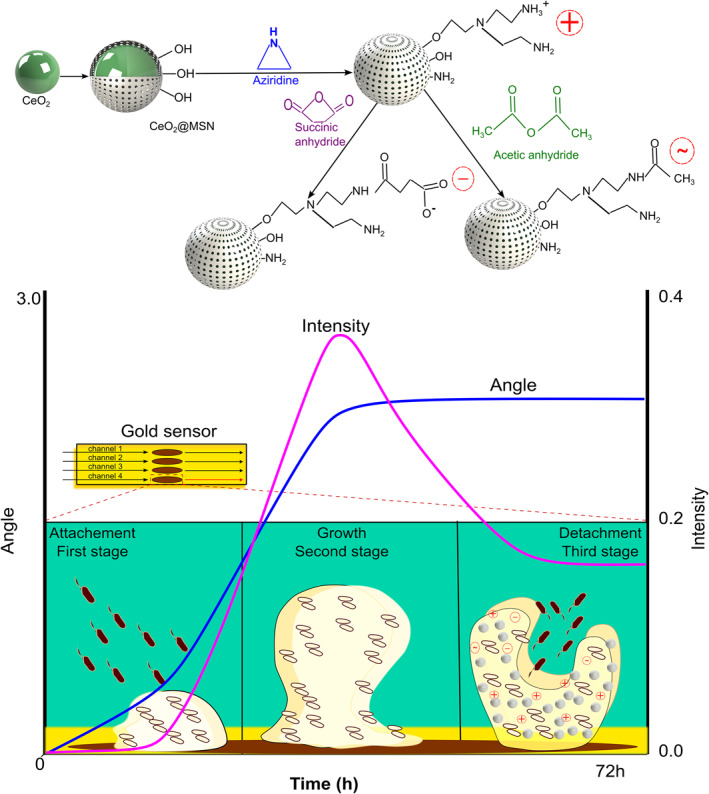

**Key points**

- Antimicrobial resistance belongs to the 10 biggest threats to global health.
- Development of new analysis platforms and drug delivery systems is of utmost importance to find innovative antimicrobial treatment modalities.
- A label‐free analysis platform for real‐time monitoring and characterization of bacterial biofilm growth stages was developed.
- The label‐free analysis platform was capable of revealing differences in the interactions between various antibacterial nanoparticles and bacterial biofilms.

## 1 INTRODUCTION

Antimicrobial resistance is considered as one of the biggest risks to public health. By 2050, the number of deaths attributed to bacterial infections will surpass the number attributed to cancer‐related diseases, reaching around 10 million people annually.^1^ Planktonic cells are the most common source of acute infections, which can be treated with antibiotics.^2^ While most environmental risk evaluations are performed on planktonic organisms, most microorganisms live and grow in highly structured aggregates called biofilms.^3^ Bacterial biofilm is a multimicrobial community composed predominantly of bacteria that is affixed to a surface and embedded in its own extracellular matrix (ECM).^4^ The ECM is composed of diverse bacterial secreted polymers, including exopolysaccharides, extracellular DNA, proteins, and amyloidogenic proteins.^3^ ^c^^,^ ^5^ Bacteria in biofilms are considered to be responsible for chronic infections.^4^ ^a^ Bacterial biofilms are thought to be responsible for 65%–80% of all infections that arise in the human body.^3^ ^b,^ ^6^ Due to its physicochemical features, the bacterial biofilm matrix functions as a barrier to antibiotics. The biofilm microenvironment with its gradients of pH, oxygen, and nutrients can affect antibiotic effects, and the existence of persisted bacteria further increases the resistance to antibiotic therapy.^5^ ^a,^ ^5^ ^b,^ ^7^ Such a lifestyle enables microorganisms to evade the immune system and may require up to 1000 times the least inhibitory dose of antibiotics in bacterial biofilms to achieve the same result compared to bacteria in suspension.^6^ Thus, antibiotic resistance and the need for efficient drug delivery into biofilms have increased, necessitating the development of new promising approaches for preventing infection spread and providing successful therapy.^8^

Nanotechnological solutions have been extensively studied for antimicrobial medication delivery. Nanoparticles (NPs) exert their effects either by functioning as a carrier for the efficient delivery of antimicrobials or by possessing intrinsic antimicrobial characteristics.^6^ ^,^ ^8^ ^b,^ ^9^ Over the past decade, the use of metal and metal‐based NPs such as Ag, Fe, ZnO, TiO_2_, CeO_2_, or SiO_2_ has expanded in many applications, including medicine as anti‐bacterial agents.^7^, ^10^ This is because they possess non‐specific antibacterial mechanisms that may prevent antibiotic resistance, increase the spectrum of antibacterial action, and prevent biofilm formation.^11^ Recently, numerous biomedical investigations have used cerium oxide‐based NPs (CeO_2_) as anticancer, antioxidant, antibacterial, antibiofilm, and anti‐inflammatory agents, as well as bio scaffold agents.^10^ ^c,^ ^12^ However, CeO_2_ NPs aggregation remains a challenge for efficient use.^13^ Researchers have used surfactants, polymer coatings, dry storage, sonication, and a silica coating to prevent CeO_2_ NPs from aggregating.^14^ The most successful way has shown to cover CeO_2_ NPs with inorganic mesoporous silica (MS) as a shell to generate CeO_2_@MSNs. The biocompatibility, large surface area, high loading capacity for active pharmaceutical compounds, and easily modified surface properties make silica an attractive material for biomedical applications.^15^ To activate NPs against bacterial biofilms, they must penetrate the fluid interface, adhere to the outer surface, and permeate and accumulate into the matrix.^10^ ^a,^ ^16^ In the case of antimicrobial NPs, however, a comprehensive understanding of their pharmacokinetic and pharmacodynamic properties is essential to enhance their efficacy in combating against antimicrobial resistance, while minimizing any potential toxicity to the host.^17^ Consequently, the physicochemical characteristics of NPs (size, shape, and surface properties) can all impact their pharmacokinetic and pharmacodynamic properties.^17^ ^a^ Particularly, the surface charge of NPs (anionic, neutral, or cationic) is crucial for predicting their behavior in biofilms.^16^ ^a,^ ^18^ Some studies have already reported that *Aeruginosa* and *Staphylococcus aureus* biofilms are more easily penetrated by cationic liposomes than anionic liposomes.^19^ Similarly, in contrast to cationic particles, which have been shown to easily penetrate *Escherichia coli* biofilms, neutral and anionic quantum dots failed to permeate into biofilms.^20^ However, the mode of action of NPs in biofilms is still poorly described or understood. Therefore, the investigation of NP‐biofilm interactions may contribute to the fight against biofilm‐associated infections, as the construction of bacterial biofilms promotes antibiotic resistance, which is a major public health concern. However, studies concerning the interaction of NPs and biofilms are limited as biofilms are intricate and require the use of many techniques to determine their physiology, structure, and composition.^10^ ^a^

The surface plasmon resonance technique (SPR) has been used for biomolecular interaction studies since the 1990s.^21^ The SPR phenomena can be induced by directing visible light through a glass prism at a specific angle (θ) on a thin metal layer (usually ∼50 nm gold) that is in contact with the prism (i.e., in the so called Kretchmann configuration). This will excite the free electrons on the metal surface, which in turn induces surface plasmons that travel along the metal surface simultaneously creating an evanescent field (sensing field) that penetrates the adjacent medium in contact with the metal. The change in the angle of the incident light at which surface plasmons are excited is proportional to the change in refractive index (*n*) and thickness (*d*) of the layer adsorbed or interacting with the metal sensor surface. Traditional SPR techniques provide a very limited angular detection range, which restricts their use to biosensing assays with thin sensing layers. Therefore, traditional SPR techniques have mostly been used to study protein−protein, drug−protein, DNA or oligonucleotide hybridization, and other biomolecular interactions that is based on relatively thin layers.

**Scheme 1:**
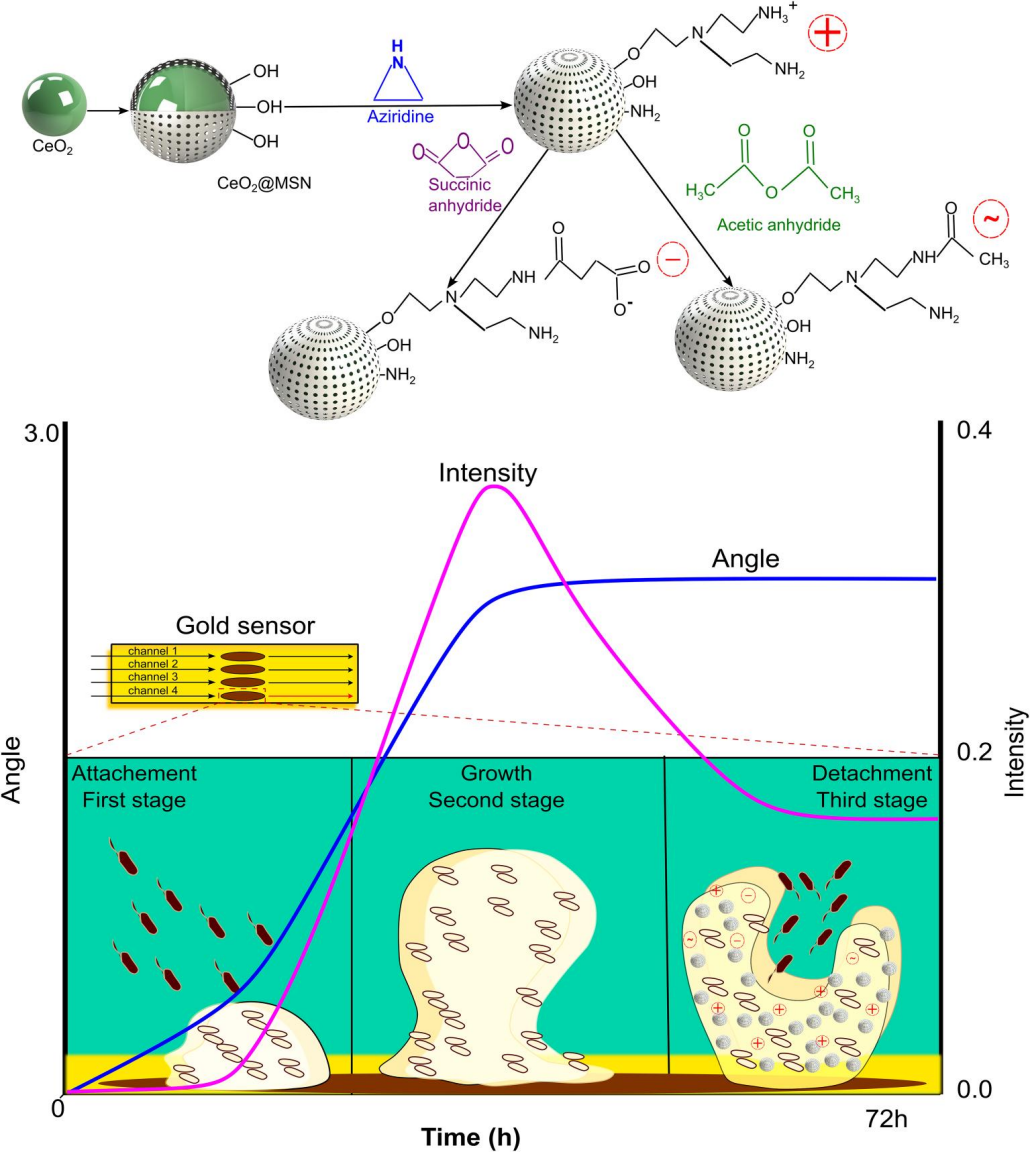
Diagrams illustrating the synthesis, surface functionalization of NPs, in situ biofilm growth monitoring in MP‐SPR for measuring NP‐biofilm interactions with MP‐SPR. Upper scheme depicts the synthesis of CeO_2_, coating CeO_2_ with an MSN layer (CeO_2_@MSN), and then modifying the surface of CeO_2_@MSN with different surface functionalizations, where (+) denotes a positive net surface charge, (−) a negative and (∼) a near‐neutral net surface charge at neutral pH. The lower scheme illustrates growing of *Staphylococcus aureus* bacterial biofilm in situ in flow within the MP‐SPR instrument and simultaneously monitoring and detecting biofilm growth phases. The blue line depicts the ΔPAP and the orchid line the ΔPMI responses measured as a function of time with MP‐SPR during bacterial biofilm growth. MP‐SPR, multi‐parametric surface plasmon resonance; NPs, nanoparticles; ΔPAP, peak angular position; ΔPMI, peak minimum intensity.

The unique multi‐parametric surface plasmon resonance technique (MP‐SPR), which also utilizes the SPR phenomena, provides a much wider angular scan range compared to traditional SPR techniques. The wider angular scan range of MP‐SPR enables the use of sensing layers with thicknesses up to several micrometers.^21^, ^22^ This has opened new opportunities to utilize the MP‐SPR in applications that have not previously been accessible for traditional SPR techniques. Recently, novel real‐time label‐free analysis platforms based on the MP‐SPR technique have been developed for measuring specific cell responses with small molecules/drugs^23^; for differentiating between major G‐protein coupled receptor cell signaling pathways when cells are stimulated with specific ligands for each pathway^24^ and for monitoring cell uptake kinetics and efficacy of various NPs.^25^ These studies have collectively shown that the MP‐SPR signal response is a result of the combination of morphological changes in the cell layer, such as cell spreading or contraction, and stimulant buildup in the cells. Thus, the MP‐SPR response should reflect the structure of bacterial biofilms during growth, and NP interactions with the bacterial biofilm should be dependent on the translocation of NPs through the biofilm. The MP‐SPR technique also has numerous advantages over conventional techniques (i.e., confocal microscopy and flow cytometry) because it enables monitoring interactions and translocation of NPs in the biofilm in real‐time; it does not require any use of fluorescent labels; it can perform continuous real‐time measurements for up to 72 h; it provides information on biofilm growth stages and biofilm properties; and NP interactions with the biofilm in a single workflow which do not require any washing steps or any other interruptions during the measurements.^26^ Considering the above‐mentioned reasons, bacterial biofilms in this study were grown in situ in flow in the MP‐SPR instrument (Scheme 1), while simultaneously monitoring the biofilm growth stages. This was immediately followed by monitoring CeO_2_@MSNs‐bacterial biofilm interactions with particles of different surface charges (Scheme 1). To our knowledge, this study demonstrates for the first time how real‐time label‐free MP‐SPR measurements enable a detailed analysis of the growth stages and properties (i.e., thickness and refractive index) of bacterial biofilms. Furthermore, this study also shows for the first time the utilization of the MP‐SPR technique for measuring real‐time kinetics of NP interactions with bacterial biofilms without the use of labeling agents.

## 2 RESULT AND DISCUSSION

### 2.1 Synthesis of ceria core@silica shell NPs (CeO_2_@MSNs)

**FIGURE 1:**
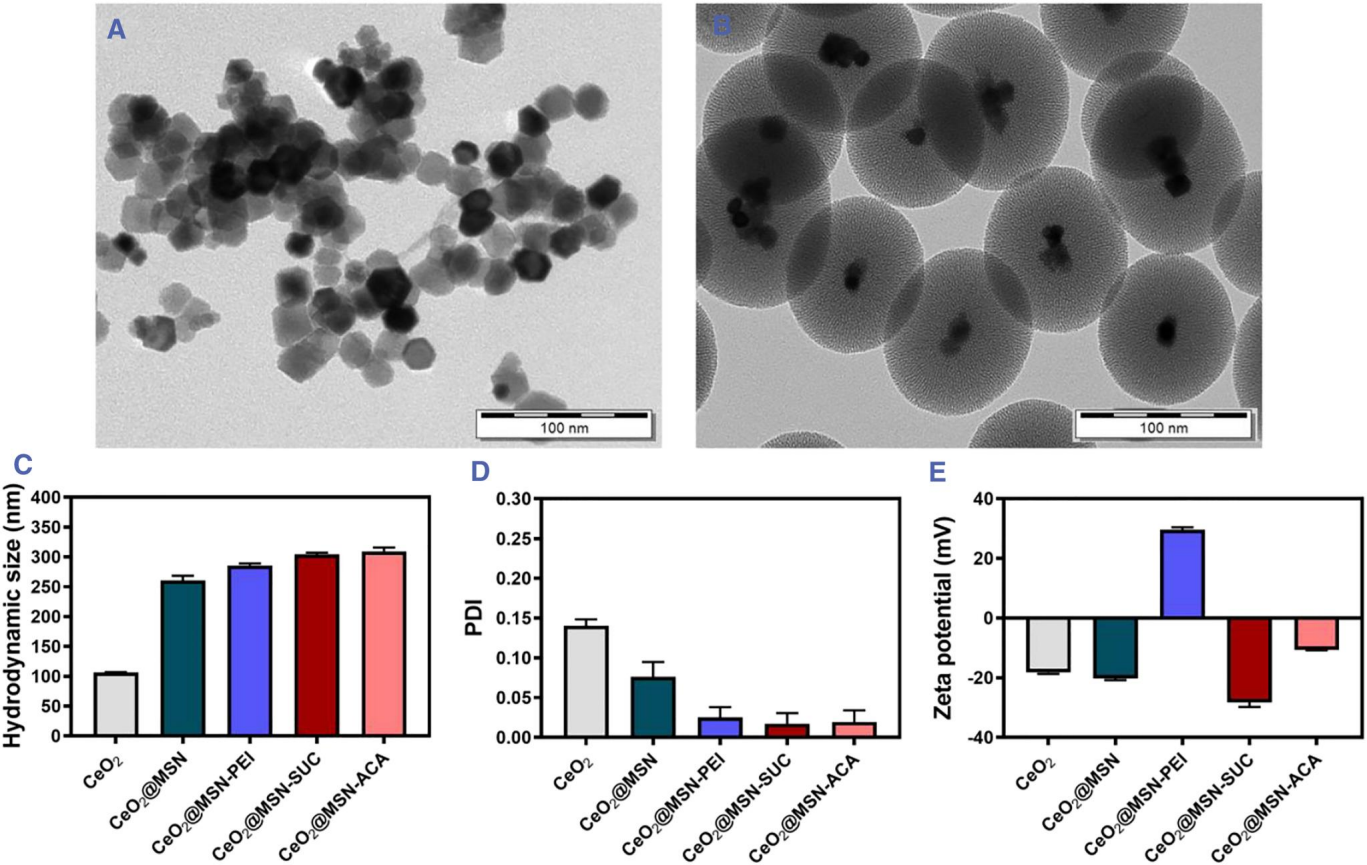
Characterization of nanoparticle morphology and surface functionalization using TEM and DLS. (A) TEM image of CeO_2_ NPs (scale bar 100 nm). (B) TEM image of CeO_2_@MSNs (scale bar 100 nm). (C) Hydrodynamic size, (D) PDI and (E) ζ‐potential of CeO_2_, CeO_2_@MSN, CeO_2_@MSN‐PEI, CeO_2_@MSN‐SUC and CeO_2_@MSN‐ACA at pH 7.2. DLS, dynamic light scattering; NPs, nanoparticles; PDI, polydispersity index; TEM, transmission electron microscopy.

The core@shell CeO_2_@MSNs were successfully produced, as shown in Figure 1A,B. Although CeO_2_ as metallic NPs has inherent antibacterial characteristics, the aggregation tendency of CeO_2_ NPs, due to their high surface‐to‐volume ratio and lack of an intrinsic colloidal stability mechanism, limits their use.^27^ The surface modification with a MS shell can improve the dispersibility, colloidal stability and even biocompatibility of CeO_2_ NPs, while also acting as a reservoir for inserting molecular antibacterial agents to provide antibacterial activity.^28^ Furthermore, silanol groups on the surface of the silica layer act as anchoring moieties for covalent binding of different functional groups to tune the surface properties of CeO_2_@MSNs while preserving the original structure of the particles. This enables the evaluation of the importance of various surface charges of the synthesized CeO_2_@MSNs in terms of their interactions with biofilms.

Initially, CeO_2_ NPs were produced using a previously described protocol with minimal modifications.^28^ As depicted in the transmission electron microscopy (TEM) image in Figure 1A, the average size of the synthesized CeO_2_ was approximately 28 ± 2 nm. Then, as depicted in Figure 1B, an MSN layer with a uniform thickness was formed over the CeO_2_ core, which caused the average size of CeO_2_@MSN to increase to around 190 ± 12 nm. As the mesoporous shell was added, this enhanced the colloidal stability of CeO_2_ containing NPs consequently leading to well‐dispersed NPs, which was also reflected in the small polydispersity index (PDI) value (Figure 1D). In addition, the synthesized CeO_2_@MSNs were modified by hyperbranched polyethylene imine (PEI) to generate additional anchoring sites, that is, for secondary coating, and to produce CeO_2_@MSN‐PEI. PEI coating gives CeO_2_@MSNs a highly positive surface charge, which increases their dispersibility and stability in physiological environments.^29^ CeO_2_@MSN‐PEI were further derivatized using succinic anhydride (SUC) to cap the terminal (primary) amino groups with carboxyl groups, which resulted in a highly negative surface charge. In contrast, acetic anhydrite capping (ACA) resulted in a surface charge within the neutral region, as illustrated in Scheme 1.^30^ The dynamic light scattering (DLS) results in Figure 1C revealed that the hydrodynamic size of synthesized CeO_2_ and CeO_2_@MSNs were 106.6 ± 0.6 and 261 ± 8 nm, respectively. The corresponding PDI values shown in Figure 1D were 0.1 ± 0.01 and 0.08 ± 0.02 and confirmed that CeO_2_ and CeO_2_@MSNs form homogeneous dispersions. In addition, the hydrodynamic size obtained for CeO_2_@MSN‐PEI, CeO_2_@MSN‐SUC, and CeO_2_@MSN‐ACA was 286 ± 3, 305 ± 3, and 310 ± 6 nm, respectively, with corresponding PDI values of 0.03 ± 0.01, 0.02 ± 0.01, and 0.02 ± 0.01 (Figure 1C,D). The DLS results demonstrated that the size of CeO_2_ NPs increased after coating with a mesoporous shell, and that the size of CeO_2_@MSNs further increased after additional surface modifications with PEI, SUC, and ACA. This indicated a successful coating of the silica layer on CeO_2_ NPs and consequent surface modification of the silica layer. The DLS analysis revealed complete re‐dispersibility of particles and a narrow particle size distribution, as shown by the low PDI values. DLS measures the hydrodynamic size, which is impacted by the hydration layer that forms on particles in solution. Therefore, particle sizes determined by DLS are typically significantly larger than those reported by TEM.^31^ Furthermore, the colloidal stability of the synthesized CeO_2_@MSN‐PEI, CeO_2_@MSN‐SUC, and CeO_2_@MSN‐ACA in tryptic soy broth (TSB) as bacterial medium was confirmed by DLS measurements. These DLS measurements showed only a modest increase in hydrodynamic size, while the PDI remained small, which confirmed the monodispersity of CeO_2_@MSN‐PEI, CeO_2_@MSN‐SUC, and CeO_2_@MSN‐ACA in bacterial culture media (Figure S1A,B).

ζ‐potential measurements for the synthesized CeO_2_ core and CeO_2_@MSNs with various surface modifications were carried out to confirm that the CeO_2_@MSNs were successfully functionalized. Figure 1E demonstrates that the CeO_2_ core had a negative ζ‐potential value (−18.3 ± 0.4 mV) since the pH of 7.2 of the buffer solution the measurements were performed in was greater than the isoelectric point of the CeO_2_ core. This caused the absolute value of the ζ‐potential of the particle surface to increase, thereby preventing attraction between particles.^32^ The ζ‐potential value of CeO_2_@MSNs remained negatively charged at pH 7.2 (−20.5 ± 0.5 mV) due to the acidic Si–OH groups of the silica coating.^28^ Moreover, the ζ‐potential of CeO_2_@MSNs became highly positive (+30 ± 1 mV) after being grafted with PEI, while consecutive functionalization with SUC turned the ζ‐potential value highly negative (−28 ± 2 mV), and closer to neutral with ACA functionalization (−10.6 ± 2.0 mV) at pH 7.2. The changes in the ζ‐potential values showed a successful surface modification of CeO_2_@MSNs with amino, carboxy, and acyl groups. The ζ‐potential values of CeO_2_@MSN‐PEI, CeO_2_@MSN‐SUC, and CeO_2_@MSN‐ACA were also confirmed in bacterial growth conditions, and were shown to correlate with the ζ‐potential values measured in the buffer with pH 7.2 (Figure S1C).^29^ ^,^ ^30^ ^b^

### 2.2 MP‐SPR measurements and optical modeling of biofilm growth and subsequent interactions with differently charged NPs

**FIGURE 2:**
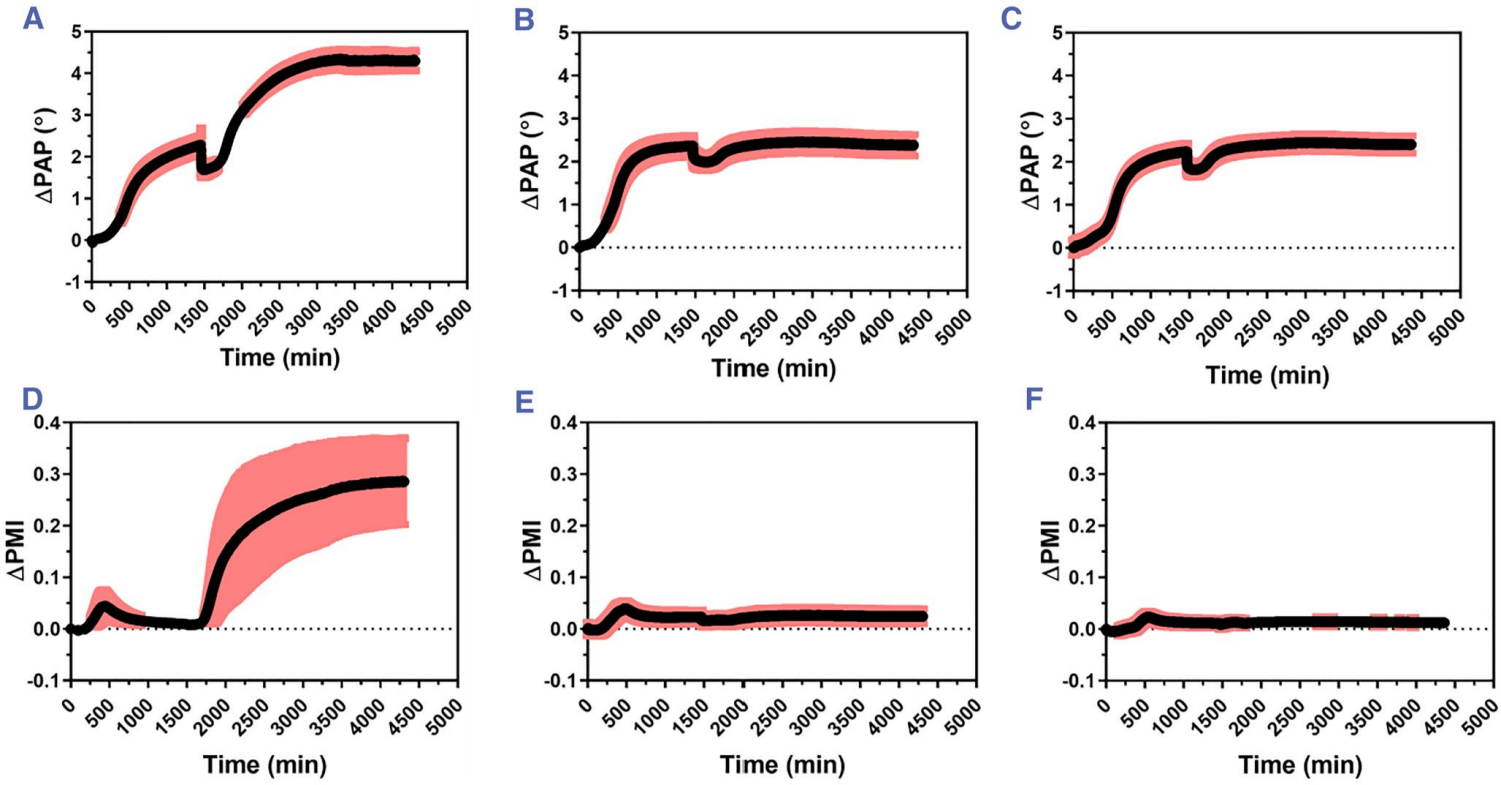
Average of real‐time MP‐SPR responses of the ΔPAP (upper graphs) and the ΔPMI (lower graphs) during *Staphylococcus aureus* bacterial biofilm growth (time: 0–1300 min) and interaction of differently charged NPs (from *t* = 1300 min and onwards). (A) ΔPAP and (D) ΔPMI of biofilm growth and interaction with CeO_2_@MSN‐PEI (*N* = 3). (B) ΔPAP and (E) ΔPMI of biofilm growth and interaction with CeO_2_@MSN‐SUC (*N* = 3). (C) ΔPAP and (F) ΔPMI of biofilm growth and interaction with CeO_2_@MSN‐ACA (*N* = 3). MP‐SPR, multi‐parametric surface plasmon resonance; NPs, nanoparticles; ΔPAP, peak angular position; ΔPMI, peak minimum intensity.

Figure 2 depicts the MP‐SPR peak angular position (ΔPAP) and peak minimum intensity (ΔPMI) responses as a function of time during the in situ formation of *S. aureus* bacterial biofilms in the MP‐SPR flow channel and subsequent interactions between differently charged CeO_2_@MSNs and the bacterial biofilms. Consecutive monitoring of the bacterial biofilm growth and NP‐bacterial biofilm interactions with the MP‐SPR exhibited clear ΔPAP and ΔPMI responses for each type of NP used. The positively charged NPs (CeO_2_@MSN‐PEI) displayed a unique real‐time signal profile compared to the negatively charged (CeO_2_@MSN‐SUC) and neutral (CeO_2_@MSN‐ACA) NPs.

#### 2.2.1 MP‐SPR ΔPAP responses during biofilm growth

The growth of the *S. aureus* bacterial biofilms was monitored as a function of time after injecting the bacterial solution into the MP‐SPR flow channels. The ΔPAP responses showed a slow initial increase within the first 200 min. This indicates that only a small proportion of bacterial cells initially adhered to the gold sensor, and that an irreversible attachment and a slow division of these bacteria occurred during the first 200 min (Figure 2A–C). After this lag phase, the ΔPAP responses exhibited a two‐phased signal increase: a rapid signal increase between 200 and 600 min and a slower increase and leveling out of the signal between 600 and 1300 min, reaching a maximum ΔPAP signal response of around 2500 mdeg. The rapid increase in the ΔPAP responses within 200–600 min showed that a significant mass was deposited on the sensor surfaces during this time, which indicates that the biofilms have entered the maturation stage and cells divide rapidly during this period.^33^ The slow increase or leveling out of the ΔPAP responses after 600 min indicated that the biofilms stopped growing at this stage and reached their final thicknesses under these experimental circumstances.

#### 2.2.2 MP‐SPR ΔPMI responses during biofilm growth

In addition to ΔPAP, the ΔPMI parameter during biofilm growth was also evaluated to assess if it would provide additional supporting information for the results obtained by measuring the ΔPAP. The ΔPMI parameter is the dynamic intensity shift of the SPR peak minimum and can be extracted when the full SPR spectra is measured.^24^ As shown in Figure 2D–F, during the growth of the *S. aureus* bacterial biofilms (*t* = 0–1300 min) the ΔPMI responses did not initially change during 200 min. After this the ΔPMI response increased rapidly and reached a maximum of 0.03–0.04 at around 400–500 min, followed by a gradual decrease and leveling out between 500 and 1300 min. The rapid increase in the ΔPMI response after the lag time is most likely due to light scattering from small colonies of bacteria that form and proliferate on the sensor surface over time.^33^ At the same time the bacteria also start to produce the ECM. This is also indicated by the fact that after reaching the maximum value, the ΔPMI response decreased and returned nearly to its initial value or levels out, which reflects that the biofilm became a flat uniform layer. Interestingly, the time point when the ΔPMI response reached the maximum value coincided with the time when the ΔPAP response had the steepest slope, while the time point when the ΔPMI response leveled out coincided with the time when the ΔPAP response also started to level out. Thus, the ΔPAP responses directly reflect the thickness/mass or the optical density of the biofilm, while the ΔPMI responses can be used to identify the different growth stages of the biofilm. In other words, during the lag phase where bacteria are dividing slowly the ΔPAP increases only slightly, while the ΔPMI remains close to zero. Then, during the first maturation phase where bacteria form colonies and start to produce the ECM, both the ΔPAP and ΔPMI responses increases rapidly. This is then followed by a second maturation phase where bacteria colonies and ECM start to fuse and form a homogeneous biofilm. The onset time for the second maturation phase is indicated by the point where the ΔPAP response reaches its highest slope and the ΔPMI response reaches its maximum value. Finally, when the ΔPAP and ΔPMI response levels out, then the biofilm growth has reached its end.

#### 2.2.3 Optical modeling of biofilm properties

**FIGURE 3:**
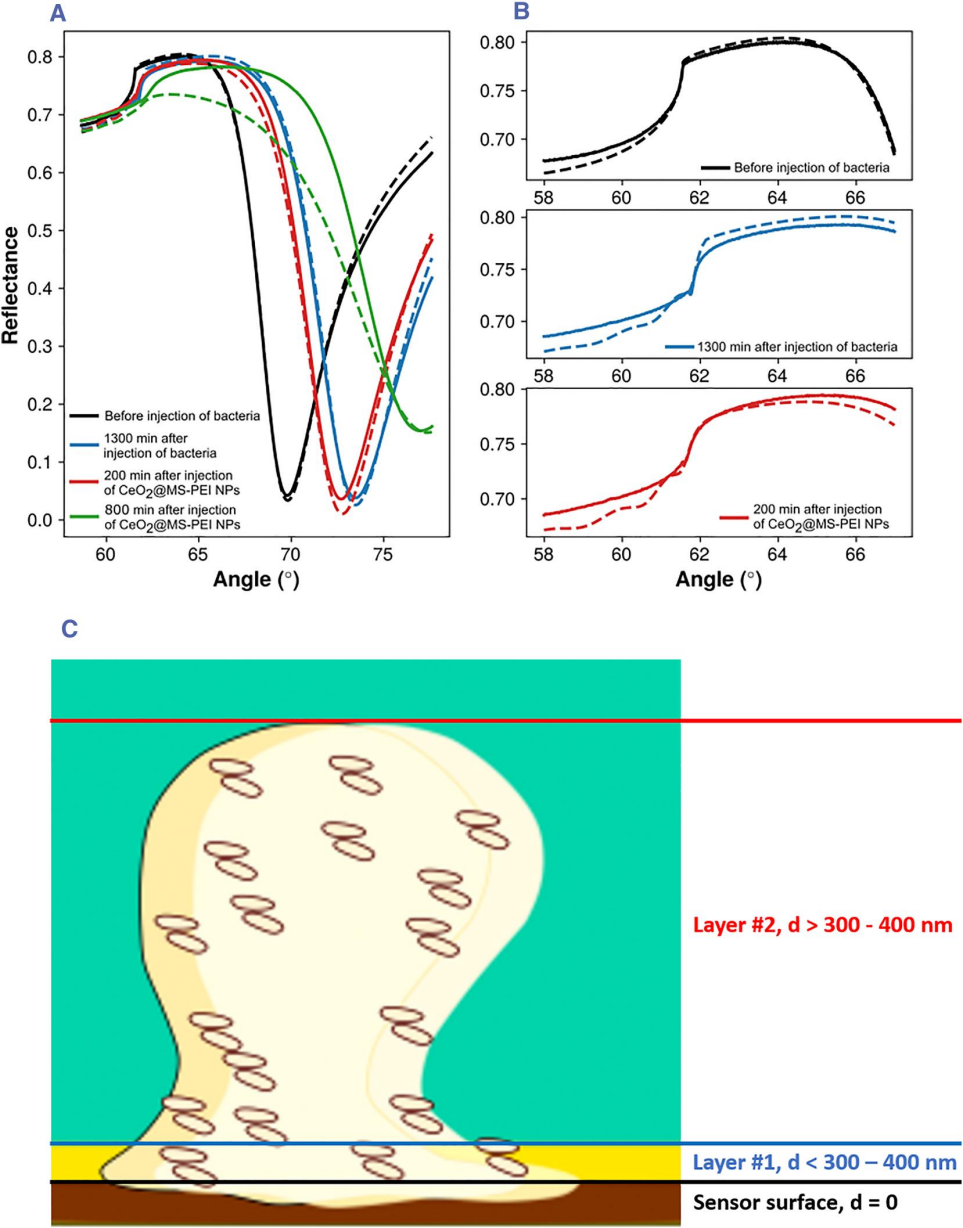
(A) MP‐SPR reflectance spectra 5 min before the injection of bacteria (black), *t* = 1300 min after the start of the injection of bacteria (blue), 200 min (red, time = 1500 min in Figure 2A) and 800 min (green, *t* = 2100 min in Figure 2A) after the start of the injection of CeO_2_@MSN‐PEI NPs. (B) Total internal reflection angle region of the data shown in panel (A). All data are for the 670 nm wavelength, and dotted lines indicate the optical (Fresnel) fits to the measured data. (C) Schematic representation of the sample layers used in the optical modeling of the full SPR reflectance spectra. MP‐SPR, multi‐parametric surface plasmon resonance; NPs, nanoparticles.

The MP‐SPR data at the end of the biofilm growth (time = 1300 min) were analyzed by fitting the full SPR reflectance spectra (Figure 3) to a model consisting of two layers. The first layer (#1) represents the accumulated mass within the thin 300–400 nm thick evanescent field region above the sensor surface, and the second layer (#2) represents the rest of the biofilm, that is, >300–400 nm. The first layer (#1) is dominantly linked to the ΔPAP and ΔPMI responses and is characterized by both layer thickness and refractive index. However, these parameters cannot be separated without knowing the exact relationship between the two at different wavelengths. Therefore, the layer thickness derived for the first layer does not necessarily reflect the physical dimensions. In contrast, the second layer (#2) affecting the total internal reflection (TIR) region of the reflectance spectrum can be characterized both in terms of layer thickness and refractive index, since the shape and position of the TIR region are uniquely determined by them. Figure 3 (blue curves) shows the presence of a faint waveguide peak in the TIR region that forms when a layer on the sensor is significantly thicker than the evanescent field penetration depth, that is, thicker than 300–400 nm. It can be assumed that the layer parameters for the second modeled layer #2 can be used to represent the biofilm thickness and refractive index, which were 2.7 ± 0.6 and 1.35 ± 0.01 μm for the biofilms before injection of NPs. Values for the modeling of layer #2 are presented in Table S1.

#### 2.2.4 MP‐SPR ΔPAP responses during interactions of differently charged NPs with the biofilm

When the bacterial biofilms had reached their final thicknesses, then differently charged NPs were immediately injected into the MP‐SPR flow channels to monitor NP‐bacterial biofilm interactions. The positively charged CeO_2_@MSN‐PEI exhibited an instantaneous response in the ΔPAP upon contact with the biofilm and induced a small decrease in the signal between 1300 and 1800 min. This was followed by a rapid increase and leveling out of the signal between 1800 and 4000 min, reaching a maximum ΔPAP signal response of around 4500 mdeg (Figure 2A). In the case of negatively charged CeO_2_@MSN‐SUC and neutral CeO_2_@MSN‐ACA the ΔPAP signal responses showed different ΔPAP response profiles compared to the positively charged CeO_2_@MSN‐PEI (Figure 2B,C). First, an initial decrease between 1300 and 1800 min in the ΔPAP responses upon contact of the negatively charged and neutral NPs with the biofilm was seen, similarly as was seen for the positively charged CeO_2_@MSN‐PEI. However, this was followed by rapid increases and leveling out of the ΔPAP signal responses between 1800 and 4500 min reaching maximum ΔPAP signal responses of the same level as the original biofilms, that is, 2500 mdeg. This indicates that the negatively charged CeO_2_@MSN‐SUC and neutral CeO_2_@MSN‐ACA do not interact in the same extent and strength with the biofilms compared to the positively charged CeO_2_@MSN‐PEI. Due to the penetration depth of the evanescent field in SPR, the detection depth for MP‐SPR is limited to 300–400 nm from the sensor surface. This is much smaller compared to the thickness of the biofilms. The Fresnel modeling approach (Figure 3, red lines) suggests that MP‐SPR initially detects morphological or structural changes in the lower parts of the bacterial biofilms when NPs are injected.^33^ ^b^ The initial decrease in the ΔPAP signal responses seen for all NPs used in this study can thus be attributed to such morphological or structural changes. These morphological or structural changes could cause the bottom part of the biofilm to reduce contact points with the sensor surface or loosen up the matrix structure and allow more media into the SPR detection region when the biofilms are exposed to NPs. These types of morphological or structural changes would cause a decrease in the ΔPAP signal response. However, further accumulation of NPs in the biofilm would result in a steadily increasing ΔPAP signal response that would level off when the particles reach a saturation level within the evanescent field region.^33^ ^a^ This type of action is indicated by the ΔPAP responses for the positively charged CeO_2_@MSN‐PEI for which the responses increased to a level that is much higher than the signal responses for the original biofilms. Whereas, for the negatively charged CeO_2_@MSN‐SUC and neutral CeO_2_@MSN‐ACA the signal responses remained at the levels of the initial biofilms. The ΔPAP responses of the bacterial biofilm growth stages and the NP‐bacterial biofilm interaction stages were also separated into different graphs and are presented in Figure S2.

#### 2.2.5 MP‐SPR ΔPMI responses during interactions of differently charged NPs with the biofilm

The ΔPMI response rapidly increased upon contact of the positively charged CeO_2_@MSN‐PEI with the biofilm (Figure 2D, *t* = 1300–1800 min). This was followed by a leveling out of the signal between 1800 and 4000 min, reaching a maximum ΔPMI signal response of around 0.3 (Figure 2D). In the case of negatively charged CeO_2_@MSN‐SUC and neutral CeO_2_@MSN‐ACA the ΔPMI signal responses remained essentially on the same level as for the original biofilm. The different behavior of the ΔPMI responses for the different NPs used in this study is probably due to that the ΔPMI is affected by the amount of mass on the sensor surface and the quantity of light reflected at the SPR coupling angle. Additionally, the ΔPMI is also sensitive to any absorbing or scattering surfaces within the detection depth. As a result, when more NPs accumulate in the biofilm, this would result in a progressively growing signal that would level off when the particles reach and saturate in the evanescent field region.^33^ ^b^ Compared to the ΔPMI response for the biofilms, the ΔPMI response for the positively charged CeO_2_@MSN‐PEI increased and remained at a constant high level and was not decreasing as the measurement continued. This would suggest that the positively charged CeO_2_@MSN‐PEI were penetrating the biofilm all the way close to the sensor surface. While penetrating the biofilm, the CeO_2_@MSN‐PEI started to absorb or scatter light and/or modified the biofilm structure in such a way that the biofilm structure itself also started to absorb or scatter light. This is also supported by the optical modeling (Figure 3A, red lines, *t* = 200 min and green lines, 800 min after NP injection; Table S1), which showed that there was an increase in the complex refractive index of the first (#1) layer, that is, an increase in absorption or scattering of light, during the interaction of CeO_2_@MSN‐PEI with the biofilm. This was especially seen at longer interaction times, that is, 800 min after injection of CeO_2_@MSN‐PEI, where the complex refractive index and the thickness of the first (#1) layer clearly increased (Table S1). It is worth mentioning that the poor fit of the full SPR reflectance spectra for the CeO_2_@MSN‐PEI 800 min after injection (Figure 3A, green lines) is due to the limitation of the optical Fresnel modeling. The Fresnel modeling assumes that the modeled layers are evenly distributed homogeneous layers and it does not or cannot properly consider the roughness or inhomogeneity of the modeled layer. Despite this, the optical modeling provided support for the fact that the ΔPMI response can be interpreted as an increase in the complex refractive index of the first (#1) layer and that this is connected to the penetration of CeO_2_@MSN‐PEI into the biofilm. None of the behavior in the ΔPMI response during interactions of CeO_2_@MSN‐PEI with the biofilm could be seen for the negatively charged CeO_2_@MSN‐SUC and neutral CeO_2_@MSN‐ACA. This indicates that the negatively charged CeO_2_@MSN‐SUC and neutral CeO_2_@MSN‐ACA do not interact in the same extent and strength with the biofilms compared to the positively charged CeO_2_@MSN‐PEI. This was further supported by the ΔPAP results and optical modeling. The ΔPMI responses of the bacterial biofilm growth stages and the NP‐bacterial biofilm interaction stages were also separated into different graphs and are presented in Figure S3.

#### 2.2.6 Optical modeling during interaction of NPs with the biofilms

Final remark that can be made from the optical modeling is the rounding of the TIR angle region seen as a gradual upright shift and eventual disappearance of the characteristic waveguide peak after the injections of NPs (Figure 3B, red lines). This event can be modeled as an increase in the imaginary part of the refractive index of the second layer (#2) representing the biofilm above the evanescent field region. Like ΔPMI, this can be attributed to the absorption or scattering of light in the entire biofilm. However, since this rounding event was also observed during the biofilm maturation stage, it cannot be concluded that it is exclusively due to the interactions of NPs with the biofilm. However, the ΔPMI response can be seen as a more sensitive parameter since it depends on the structural changes occurring inside the evanescent field. Nevertheless, the ability to follow time‐resolved changes in the TIR region reflects changes in the biofilm structure instead of just the limited region above the sensor surface. This is a unique property of the MP‐SPR method and should be considered when analyzing properties of biofilms or cells grown on SPR sensors in general.

#### 2.2.7 MP‐SPR ΔPAP versus ΔPMI responses

**FIGURE 4:**
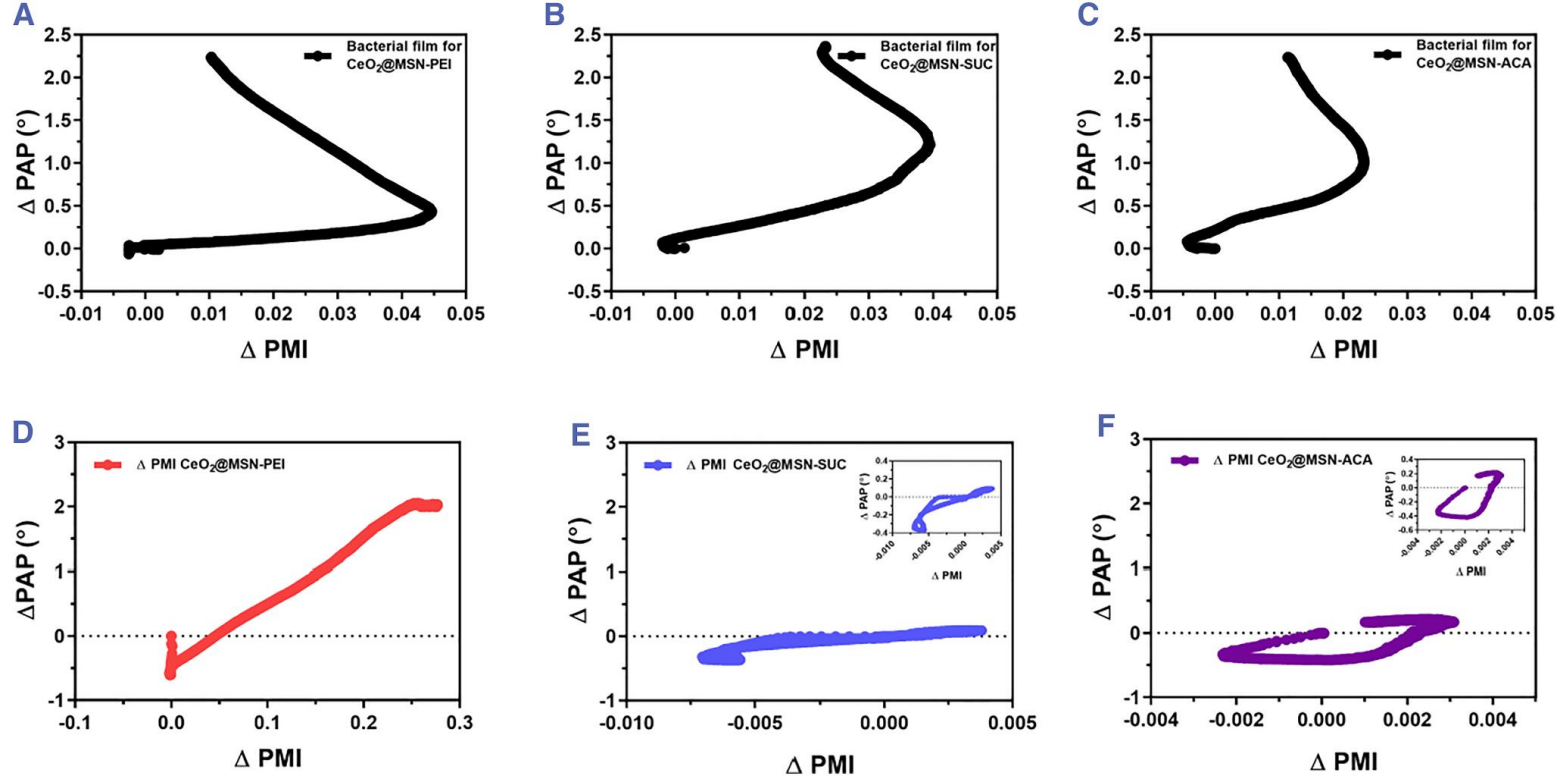
Two‐parameter SPR response profiles. ΔPAP responses are displayed against ΔPMI responses for (A–C) for bacterial biofilms in three different measurements and during biofilm interaction with (D) CeO_2_@MSN‐PEI (E) CeO_2_@MSN‐SUC (F) CeO_2_@MSN‐ACA (*N* = 3). SPR, surface plasmon resonance; ΔPAP, peak angular position; ΔPMI, peak minimum intensity.

Previous studies where MP‐SPR was used to monitor cell responses during stimulation with small molecular drugs have demonstrated that instead of analyzing ΔPAP and ΔPMI parameters separately, a plot of ΔPMI against ΔPAP responses were able to distinguish the mode of drug interaction with cells and separate various cell signaling pathways from each other.^24^ Thus, as a qualitative evaluation to understand the biofilm growth process and NP interactions with the biofilm ΔPMI–ΔPAP response profiles were also plotted for the biofilm growth measurements and for the interaction measurements of differently charged CeO_2_@MSNs with biofilms (Figure 4). The ΔPMI–ΔPAP plots for all the biofilm growth measurements (Figure 3A–C) showed a linear positive slope in the beginning which changed direction at the time point when the ECM started to form and when the biofilm matured. All the ΔPMI–ΔPAP plots for the biofilm growth measurements showed a similar pattern which indicates that the bacterial biofilms followed the same growth process and had similar structural properties, which is also supported by the optical modeling results (Figure 3, Table S1). In the case of the interactions of the differently charged CeO_2_@MSNs and bacterial biofilms, the PMI–ΔPAP plots showed two clearly distinctive response profiles (Figure 4D–F). The positively charged CeO_2_@MSN‐PEI created a straight line with a constant positive slope, while the negatively charged CeO_2_@MSN‐SUC and neutral CeO_2_@MSN‐ACA exhibited similar patterns, with a horizontal appearance. This behavior indicates that CeO_2_@MSN‐PEI has a different mode of interaction with the bacterial biofilm compared to CeO_2_@MSN‐SUC and CeO_2_@MSN‐ACA. Thus, there are some structural changes in the bacterial biofilm during penetration of CeO_2_@MSN‐PEI, while the bacterial biofilms stayed rather intact during interaction with CeO_2_@MSN‐SUC and CeO_2_@MSN‐ACA, mainly because they were not able to readily penetrate the biofilm.

### 2.3 Confocal microscopy

**FIGURE 5:**
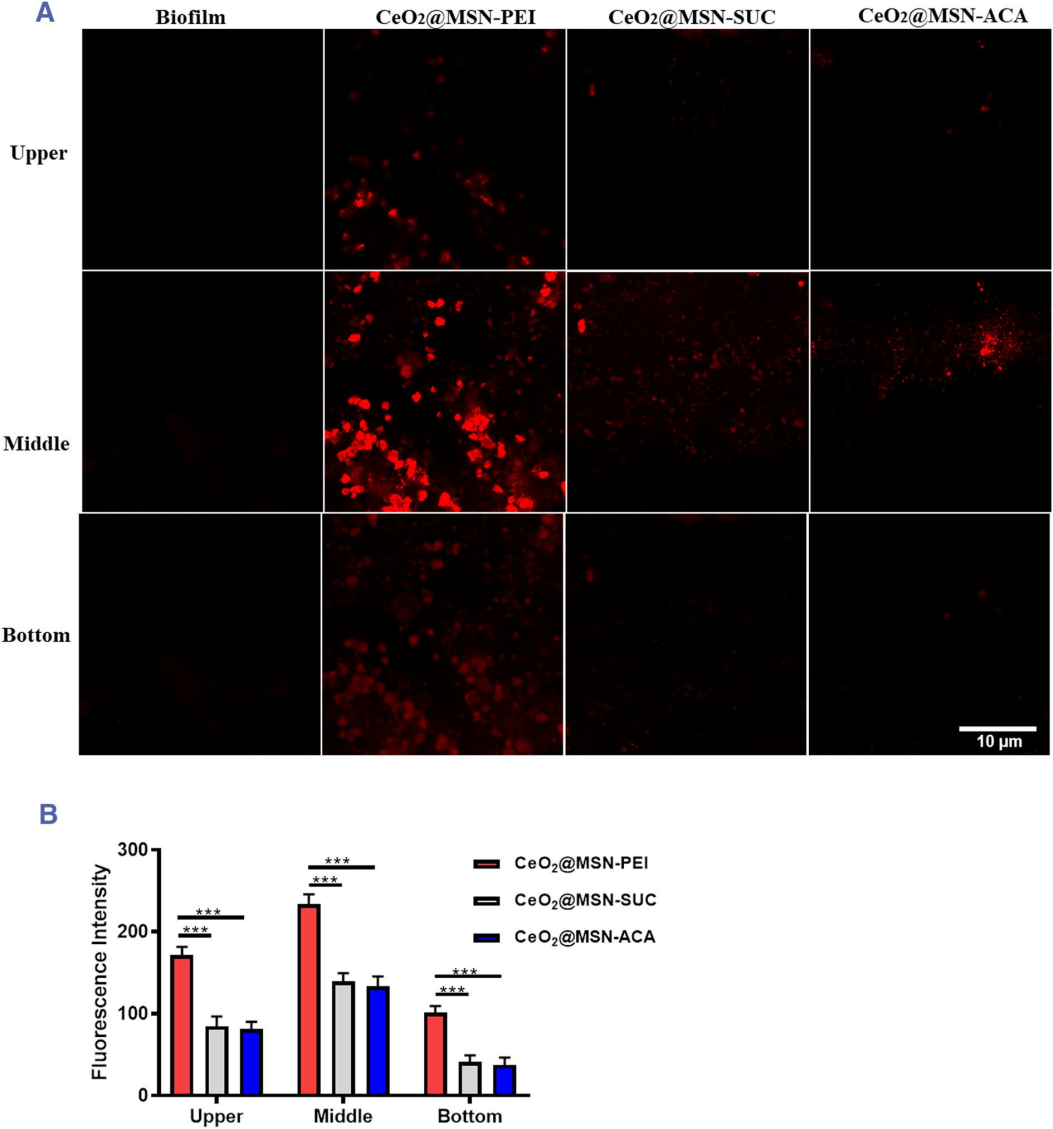
(A) Confocal microscope image stacks of plain biofilm (first column of images) and biofilm exposed with differently charged labeled CeO_2_@MSN‐PEI (second column of images), CeO_2_@MSN‐SUC (third column of images) and CeO_2_@MSN‐ACA (fourth column of images). (B) Quantitative analysis of the image stack of biofilm after exposure with differently charged labeled CeO_2_@MSN‐PEI, CeO_2_@MSN‐SUC and CeO_2_@MSN‐ACA (*N* = 3, ***P < 0.001).

Confocal laser scanning microscopy (CLSM) was used to probe the location of the NPs in the biofilms. Figure 5A shows CLSM images for a plain biofilm, and biofilms exposed to the three different NPs used in this study. Following biofilm formation in situ, labeled particles were introduced into the biofilm and Z‐stacks were obtained. The upper layer represents the uppermost layer of the biofilm, the bottom layer represents the lowermost layer of the biofilm, and the middle layer represents the middle portion of the biofilm. The positively charged CeO_2_@MSN‐PEI clearly showed the ability to penetrate the biofilm as it was detected at all depths within the biofilm and exhibited a particularly strong signal in the middle section of the biofilm.^16^ ^a^ The negatively and neutrally charged NPs exhibited a behavior that would be expected for slightly or non‐permeable NPs as they were predominant in the upper and middle layers within the biofilm.^19^ In addition, as shown in (Figure 5B), the intensity of positively charged CeO_2_@MSN‐PEI is much stronger in the upper layer of the biofilm compared to the negatively charged and neutral NPs at the top layer, which indicates that the interaction of positively charged CeO_2_@MSN‐PEI with bacterial biofilms is faster and stronger than for negatively charged and neutral NPs. Similarly, the intensity of positively charged CeO_2_@MSN‐PEI is significantly higher than that of negatively charged and neutral NPs in the middle and bottom layers of the bacterial biofilm. Based on the optical modeling (Figure 3, Table S1), the ΔPAP and ΔPMI responses arise mainly from interactions or penetration of NPs within the thin (∼300–400 nm) region of the decaying evanescent field above the sensor surface. Therefore, the accumulation of positively charged CeO_2_@MSN‐PEI on the bottom of the biofilm seen from the CLSM images correlates very well with the real‐time label‐free MP‐SPR measurements (Figure 2) and the optical modeling (Figure 3; Table S1).

## 3 CONCLUSIONS

In this study, we developed and demonstrated the use of an MP‐SPR analysis platform to evaluate the significance of NP surface properties on their interactions with bacterial biofilms. For this, we successfully synthesized and characterized CeO_2_@MSNs terminated with amine (PEI), carboxylic acid (SUC) and acyl (ACA) surface groups to render the particles positively, negatively, and neutrally charged at physiological pH. Our results showed that the MP‐SPR technique is a highly powerful platform for real‐time monitoring of bacterial biofilm growth stages and subsequent interaction kinetic studies between antibacterial NPs and bacterial biofilms. This was accomplished in a single workflow for up to at least 72 h under dynamic flow conditions and without any use of labeling agents. The MP‐SPR platform provided details on the structure and growth phases of bacterial biofilms, as well as additional insights on NP‐biofilm interactions, such as their interaction strength with the biofilm; their ability to translocate through the biofilm and the translocation time of the NPs through the biofilm. The MP‐SPR responses could be used to identify and pinpoint the time points for the different growth stages of the bacterial biofilms, which are valuable data for verifying the successful formation of bacterial biofilms. The differences in the effectiveness of the interaction between differently charged NPs and biofilms were also clearly reflected in the MP‐ SPR responses. We found that the interaction of the positively charged CeO_2_@MSN‐PEI NPs with the biofilms was much faster and stronger, and their translocation efficiency through the biofilms was much higher compared to the negatively charged CeO_2_@MSN‐SUC and near‐neutral CeO_2_@MSN‐ACA NPs. This was also confirmed with CLSM measurements. We believe that the MP‐SPR analysis platform developed in this study together with the presented results will open new opportunities and trigger new ideas to study the formation kinetics, structure, and growth mechanisms of biofilms. Especially, for studying subsequent interactions between antimicrobial agents and biofilms under precisely controlled dynamic experimental conditions and more complex study designs compared to traditional static bacterial assays.

## 4 MATERIAL AND METHODS

### 4.1 Materials

Cerium (III) nitrate hexahydrate (99% trace metals base), tetraethyl orthosilicate (TEOS), absolute ethanol, ammonium nitrate (NH_4_NO_3_), SUC, acetic anhydride (ACA), toluene, acetic acid (CH_3_COOH) and lysogeny broth (LB) were purchased from Sigma‐Aldrich, aziridine (ethylenimine) (PEI) were purchased from Menadiona, S.L., TSB was purchased from Fluka, cetyltrimethylammonium bromide (CTAB) and 3‐aminopropyltriethoxysilane (APTES) were purchased from Argos, 32% hydrogen peroxide was purchased from (EMD Millipore Corp), ammonia solution (NH_4_OH 32%) (Merck KGaA, Germany), cyanine 3 NHS ester (Cy3) were purchased from Lumiprobe.

### 4.2 Synthesis of CeO_2_ core and core@shell cerium oxide‐silica NPs (CeO_2_@MSNs)

The CeO_2_ used as the core NP was synthesized using a two‐stage procedure that included precipitation and aging stages with minor modification.^34^ The 0.26 M precursor solution was made by dissolving cerium (III) nitrate hexahydrate (99% trace metals base) in 50 mL deionized water and stirring at 70°C under reflux. With the addition of (3 M, 25 mL) ammonium hydroxide solution, the pH of the solution was raised to 8.8 and the reaction was immediately started and lasted for 5 min. For the aging of cerium hydroxide (Ce (OH)_3_) precipitate, the solution was incubated at 65°C for 20 h without stirring. During this stage, which is referred to as the aging stage, the precipitates were further dehydrated and proceeded through a dissolution–recrystallization process under ambient conditions. Following incubation, the solution was separated using a centrifuge at 10,000 rpm for 15 min at room temperature. After removing the supernatant, the pellets were washed five times with deionized water. The final product was retained in suspension in deionized water and kept at a temperature of +4°C for further use.

Sol‐gel method was used to create a shell of MS to enclose the CeO_2_ core based on published protocol with minor modification.^28^ Briefly, 1 mg/mL of CeO_2_ NP dispersion in deionized water was prepared. The CeO_2_ NP suspension was then added dropwise to the synthesis solution consisting of 4.3 mL of deionized water, 2.9 mL of 100% ethanol, and 40 mL of 32% ammonia while the solution was being sonicated. The mixture was sonicated for 30 min. Afterward, a surfactant solution was prepared by sonicating 40 mg of hexadecyltrimethylammonium bromide (CTAB) with 660 mL of milli‐Q water and 300 mL of 100% ethanol. Subsequently, the surfactant solution was added drop by drop to the synthesis mixture while it was being sonicated, and sonication was continued for an additional 30 min. Finally, 80 μL of TEOS (≥99% purity), as a precursor to silica, was added to the mixture dropwise. The reaction mixture was stirred at room temperature for 18 h. The product was collected by centrifugation at 12,000 rpm for 15 min at 18°C to eliminate any unreacted chemicals from the product. This was followed by washing twice with extraction solution containing 20% ammonium nitrate and absolute ethanol to remove extra CTAB as s surfactants template for the silica layer, and once with absolute ethanol. As a final product, CeO_2_@MSNs were stored in an ethanol dispersion at +4°C for future usage.

### 4.3 Surface modification of cerium oxide‐silica as core@shell NPs (CeO_2_@MSNs)

The surface modification of the synthesized CeO_2_@MSNs was performed with the aid of surface polymerization of aziridine to yield hyperbranched PEI to obtain highly positively charged CeO_2_@MSN‐PEI. The CeO_2_@MSN‐PEI were obtained by redispersing 100 mg of pre‐synthesized CeO_2_@MSNs in 10 mL toluene under sonication. Then, 10.4 μL of acetic acid and 400 μL of aziridine were added to the CeO_2_@MSNs suspension and the reaction was continued under constant stirring at 50°C overnight. The next day, the CeO_2_@MSN‐PEI were collected as a final product by centrifugation and washed and stored in ethanol. Then, the obtained CeO_2_@MSN‐PEI were further functionalized using SUC to produce highly negatively charged CeO_2_@MSN‐SUC. This was conducted by redispersing 25 mg of CeO_2_@MSN‐PEI in 12.5 mL absolute ethanol (2 mg/mL), followed by adding 2.5 mL of freshly prepared SUC stock solution (12.5 mg/2.5 mL) in absolute ethanol under stirring and let the reaction continue overnight at room temperature. Further, the pre‐synthesized CeO_2_@MSN‐ PEI were further functionalized to produce neutrally charged CeO_2_@MSN‐ACA. Twenty five mg of CeO_2_@MSN‐PEI were redispersed in 25 mL ethanol (1 mg/mL) under sonication. Then, 5 µL of acetic anhydride solution was added to the NP suspension under stirring, and the reaction was continued overnight at room temperature. The washing procedure in all three different surface modifications of CeO_2_@MSNs was done with ethanol twice, and the final surface functionalized NPs were collected using 12,000 rpm centrifugation for 15 min at 18°C.

### 4.4 Synthesis of CY3‐labeled CeO_2_@MSNs

The different surface functionalized of CeO_2_@MSN‐PEI, CeO_2_@MSN‐SUC, and CeO_2_@MSN‐ACA NPs were labeled with cyanine Cy3 for confocal microscope examination. Labeling was performed by taking 1 mg of surface‐modified NPs and dispersing them in dimethylformamide (1 mg/mL under sonication), then 15 μL of freshly made Cy3 solution (120 μg/mL in dimethylformamide) was added to the NP suspensions and stirred overnight in dark and at room temperature. The next day, Cy3‐labeled NPs were collected by centrifugation at 13,500 rpm for 10 min at room temperature, followed by washing twice to remove unreacted dye and drying in a vacuum drier for 20 min for further usage.

### 4.5 Preparation of bacterial cell and buffer solution (biofilm formation)

*Staphylococcus aureus* (ATCC 25923, *S. aureus*) was kept at −80°C and used as the Gram‐positive bacterium.

*Staphylococcus aureus* was cultivated aerobically at 37°C on a LB agar plate. A single colony of the bacterial cell was isolated, placed in 50 mL falcon tubes containing 10 mL LB media, and incubated for 16 h at 37°C with 250 rpm shaking. After incubation, *S. aureus* bacterial cultures were centrifuged for 5 min at 3750 rpm to remove the supernatant. The bacterial pellet was resuspended in fresh PBS, and the bacteria were counted using a cell density meter (Ultro‐spec10) at 600 nm and adjusted to 10^7^ CFU mL^−1^ in TSB for subsequent studies.

### 4.6 Monitoring NP‐bacterial film interactions using MP‐SPR

The SPR experiments were carried out using a Bionavis MP‐SPR 200 equipment (Bionavis).^35^ The gold‐coated SPR sensor surfaces were supplied from the producer of the MP‐SPR 200 device (Bionavis). Bacterial cells were seeded on gold‐coated SPR sensor surfaces in situ in the instrument at 37°C by injecting the bacterial cells through four separate MP‐SPR flow channels under constant flow rate (50 μL/min) for 24 h which allowed to develop confluent biofilms on the sensor surface prior to monitoring NP interactions with the biofilms. After injecting the NPs over the biofilm, the SPR responses were monitored for 48 h at 37°C or according to an optimized time, which would allow to detect the trafficking of the NPs through the biofilm.

### 4.7 Cleaning of gold coated SPR sensors

The gold coated SPR sensors (BioNavis) for new experiments were washed in a mixture composed of 1 part 32% hydrogen peroxide (EMD Millipore Corp), 1 part 32% ammonia solution (Merck KGaA), and 5 parts DI water. After heating the cleaning solution to the boiling point for at least 5 min, the sensor was rinsed with deionized water and 70% ethanol solution. After cleaning, the sensors were immediately inserted into the MP‐SPR instrument to start the measurements.

### 4.8 Characterization of synthesized NPs

The ζ‐potential, hydrodynamic size, and PDI of NPs were investigated by DLS (NanoZS, Malvern Instruments Ltd.). NPs were diluted in DI water and bacterial medium (TSB) at a concentration of 0.1 mg/mL to assess their size and PDI. ζ‐potential measurements were used to determine the surface charges of the NPs. ζ‐potential measurements were performed with a NP concentration of 0.1 mg/mL in either a 25 mM HEPES buffer (pH = 7.2, 25 mM HEPES FREE ACID) or bacterial medium (TSB). TEM (JEM‐1400 Plus TEM, JEOL Ltd.) was utilized to determine the size and morphology of the NPs by dispersing 5 μL of NPs at a concentration of 0.1 mg/mL on carbon‐coated copper grids (200 mesh; Ted Pella, Inc.), and then allowing them to dry in air prior to imaging.

### 4.9 Confocal microscopy

First, by injecting the bacterial cells through four different MP‐SPR flow channels at a constant flow rate (50 μL/min) for 24 h, bacterial cells were seeded on gold‐coated SPR sensor surfaces in situ in the instrument at 37°C. This allowed for the development of confluent biofilms on the sensor surface before NP interactions with the biofilms were observed. Then, labeled NPs were injected immediately at a concentration of 100 μg/mL through the SPR flow channels (50 μL/min) into the biofilm for 48 h in the dark. The SPR sensor was removed from the instrument and dried in the dark. Later, the gold coated SPR sensor with the biofilm exposed to NPs was placed upside down in a glass bottom well plate (IBL Baustoff + Labor GmbH) for confocal microscopy examination. Imaging was performed using a Zeiss LSM880 laser‐scanning confocal microscope with a 20×/0.8 objective and ZEN black software ver. 2.3 SP1 (Zeiss GmbH). Excitation was done using a 543 nm laser, and emission was collected at 548–644 nm. Three random biofilm positions along the positions of the flow channels where NPs were exposed to the biofilm were selected for imaging and quantitative analysis.

### 4.10 Data analysis

SPR data were retrieved using the SPR Navi Data Viewer (version 4.3.3) program (Bionavis Ltd.). The moment of sample injection was selected as the zero‐time point, at which both time and SPR response were set to zero. OriginPro (version b9.5.5.409) (OriginLab Corporation) and GraphPad Prism (version 8.4.1) were utilized for additional data analysis (GraphPad Software). All real‐time cell biofilm and NP‐biofilm interaction responses were adjusted for background by removing any bulk response induced by the reference sample in the control channel from the sample channel response.

The full SPR reflectance spectra were modeled by first fitting the Fresnel transfer‐matrix models at two wavelengths simultaneously (670 and 785 nm) for empty sensors without bacteria corresponding to the time points of 5 min before bacteria injection. For the analysis of the biofilm, time points of 1300 min after the start of the bacteria injection were modeled by first calculating the derivative of the TIR region of the data, which allowed to quantify the main waveguide peak position. Then, the derivatives of the Fresnel models with thickness and refractive indices of the second Fresnel layer (#2, corresponding to the biofilm thickness) as parameters were fitted to these data while keeping the thickness of the first layer as zero. In the second step, the data in the TIR region was used without taking the derivative to fit the Fresnel models with the thickness of the second Fresnel layer and refractive indices of the medium. This procedure was done for the two wavelengths separately. In the final step, the model was fitted to the data corresponding to the SPR PAP region, with the thickness and refractive indices of the first Fresnel layer (#1) as parameters. In this step, the refractive indices of the two wavelengths were linked using dispersion relation

$$n_{\lambda 2}=n_{\lambda 1}+\frac{dn}{d\lambda }∆\lambda$$

where *dn*/*dλ* = −0.1 × 10^−3^ nm^−1^ and Δ*λ* = 785–670 nm. The analysis for the time‐points after the injection of NPs was done by fitting the model to the data corresponding to the TIR region with complex refractive indices of the second Fresnel layer (#2) as parameters, and layer properties derived from earlier modeling as constants. Finally, the thickness and refractive index of the first Fresnel layer (#1) were established as parameters in fitting to the data corresponding to the SPR PAP region. The dispersion relation was used as described above. All Fresnel layer modeling was performed using custom scripts written in Python implementing non‐linear least squares minimization in scipy.optimize package.

## AUTHOR CONTRIBUTIONS

Rawand A. Mustafa: Conceptualization; Methodology; Investigation; Visualization; Data curation; Formal analysis; Writing –original draft; Writing – review & editing. Petteri Parkkila: Visualization; Software; Formal analysis; Writing – review & editing. Jessica M. Rosenholm: Supervision; Conceptualization; Resources; Funding acquisition; Writing – review & editing. Hongbo Zhang: Supervision; Conceptualization; Project administration; Resources; Funding acquisition; Writing – review & editing. Tapani Viitala: Supervision; Conceptualization; Project administration; Validation; Methodology; Resources; Funding acquisition; Writing – review & editing.

## CONFLICT OF INTEREST STATEMENT

Hongbo Zhang is an executive editor for *Smart Medicine* and was not involved in the editorial review or the decision to publish this article. All authors declare that there are no competing interests.

## ETHICS STATEMENT

No matter originating from animals nor any human subjects and/or animals were used in the experiments of this study.

## Supporting information

Supporting Information S1
