# Supplementary material for: Monitoring silica core@shell nanoparticle‐bacterial film interactions using the multi‐parametric surface plasmon resonance technique
Source: Smart Med. 2023 Jun 26;2(3):e20230012. doi: 10.1002/SMMD.20230012 (PMC11236032; doi:10.1002/SMMD.20230012)
Supplement: Supplementary file 1 — Supporting Information S1 [file SMMD-2-e20230012-s001.docx]

# Monitoring silica core@shell nanoparticle-bacterial film interactions using the multi-parametric surface plasmon resonance technique

Rawand A. Mustafa^1^, Petteri Parkkila^2^, Jessica M. Rosenholm^1^, Hongbo Zhang^1,3,4^, Tapani Viitala^1,3,^*

^1^ Pharmaceutical Sciences Laboratory, Faculty of Science and Engineering, Åbo Akademi University, 20520 Turku, Finland

^2^ Division of Nano and Biophysics, Department of Physics, Chalmers University of Technology, 412 96 Gothenburg, Sweden

^3^ Drug Research Program, Division of Pharmaceutical Chemistry and Technology, Faculty of Pharmacy, University of Helsinki, 00014 Helsinki, Finland

^4^ Turku Bioscience Centre, University of Turku and Åbo Akademi University, 20520 Turku, Finland

# Supporting Information


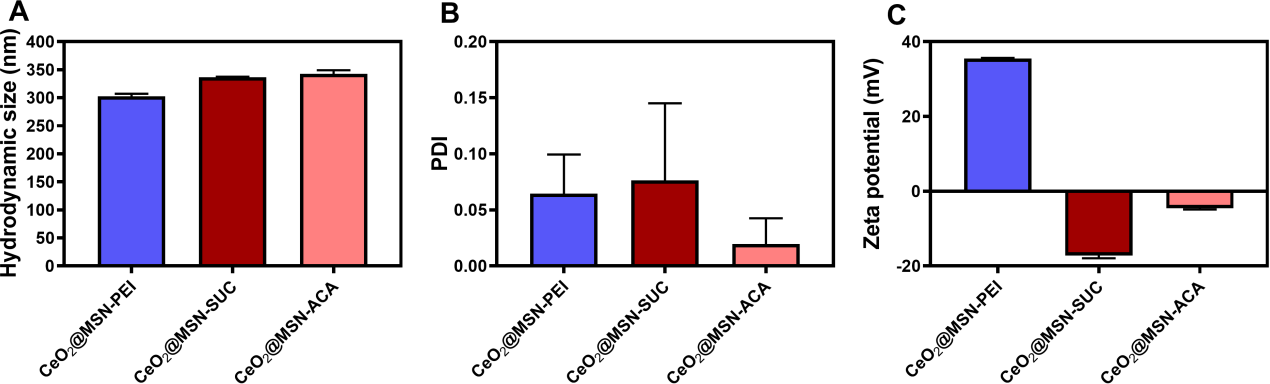


**Figure S1.** Characterization of CeO2@MSNs with different surface functionalization in bacterial culture media (TSB) using DLS. (A) Hydrodynamic size, (B) Poly dispersity index (PDI) and **(C)** ζ-potential of CeO2@MSN-PEI, CeO2@MSN-SUC, CeO2@MSN-ACA.


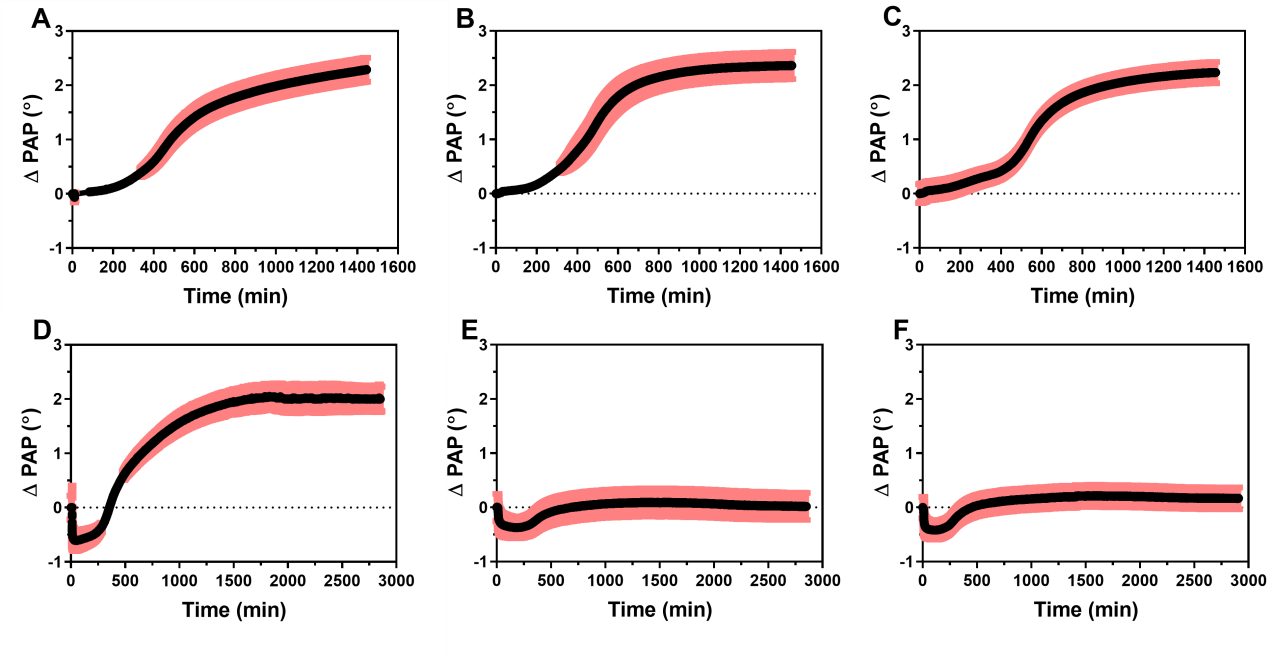


**Figure S2.** Average real-time surface plasmon resonance (SPR) peak angular position (PAP) responses during bacterial biofilm growth (upper row, A-C) and during interaction of differently charged CeO2@MSNs with the corresponding biofilm (lower row, (D) PAP for CeO2@MSN-PEI (N = 3), (E) PAP for CeO2@MSN-SUC (N = 3) and (F) PAP for CeO2@MSN-ACA) (N = 3).


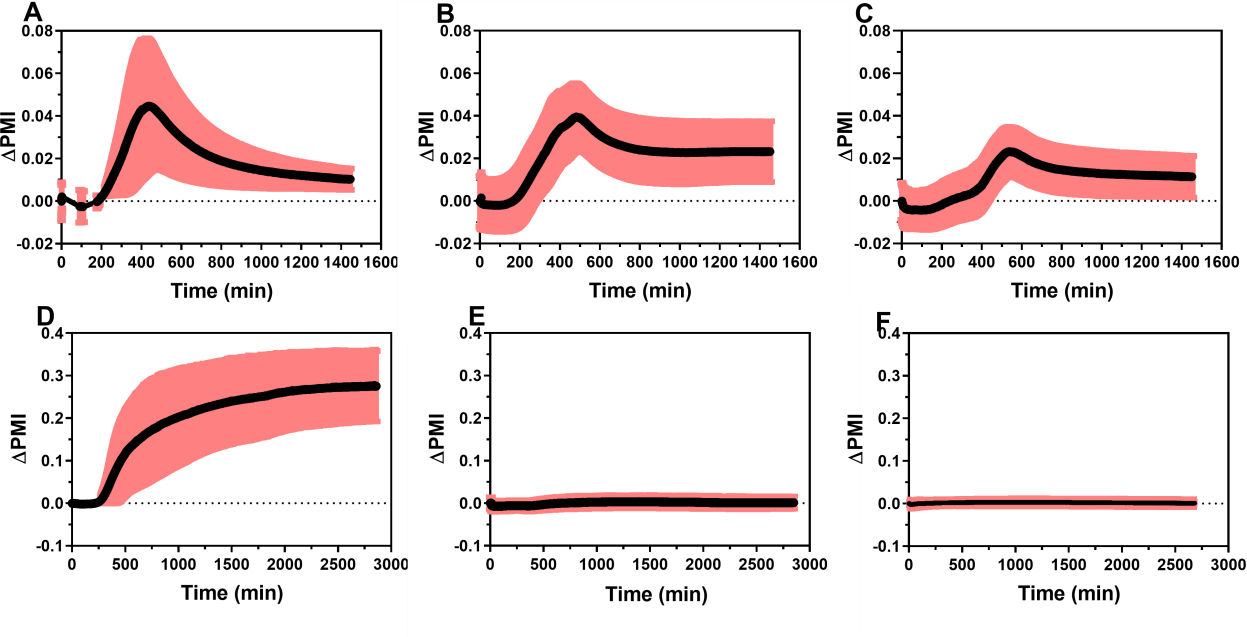


**Figure S3.** Average real-time surface plasmon resonance (SPR) peak minimum intensity (PMI) responses during bacterial biofilm growth (upper row, A-C) and during interaction of differently charged CeO2@MSNs with the corresponding biofilm (lower row, (D) PAP for CeO2@MSN-PEI (N = 3), (E) PAP for CeO2@MSN-SUC (N = 3) and (F) PAP for CeO2@MSN-ACA (N = 3).

**Table S1.** Structural parameters derived from the optical modeling of the biofilm properties using Fresnel layer modeling. *d* = thickness, *n1* = refractive index at 670 nm wavelength, *n2* = refractive index at 785 nm wavelength. Fitting parameters are highlighted in red. Additional fitting of the first model layer (#1) during NP interactions was done only for the CeO2@MSN-PEI as it was the only NP that was able to penetrate the biofilm (main manuscript, Figure 3A, green lines).

| **Experiment with CeO2@MSN-PEI** |  |  |  |
| --- | --- | --- | --- |
| 1300 minutes after injection of the bacteria | ***d (um)*** | ***n1*** | ***n2*** |
| Layer #2 | 3.38 | 1.343 | 1.343 |
| Medium |  | 1.345 | 1.344 |
| 200 minutes after NP injection | ***d* (µm)** | ***n1*** | ***n2*** |
| Layer #2 | 3.38 | 1.341 + 0.0013i | 1.340 + 0.0011i |
| Medium |  | 1.345 | 1.344 |
| 800 minutes after NP injection | ***d* (µm)** | ***n1*** | ***n2*** |
| Layer #1 | 0.155 | 1.402 + 0.0188i | 1.391 + 0.0286i |
| Layer #2 | 3.38 | 1.344 + 0.0053i | 1.343 + 0.0043i |
| Medium |  | 1.345 | 1.344 |
| **Experiment with CeO2@MSN-SUC** |  |  |  |
| 1300 minutes after injection of the bacteria | ***d* (µm)** | ***n1*** | ***n2*** |
| Layer #2 | 2.24 | 1.367 | 1.366 |
| Medium |  | 1.372 | 1.369 |
| 200 minutes after NP injection | ***d* (µm)** | ***n1*** | ***n2*** |
| Layer #2 | 2.24 | 1.359 + 0.0049i | 1.365 + 0.0017i |
| Medium |  | 1.372 | 1.369 |
| **Experiment with CeO2@MSN-ACA** |  |  |  |
| 1300 minutes after injection of the bacteria | ***d* (µm)** | ***n1*** | ***n2*** |
| Layer #2 | 2.52 | 1.348 | 1.347 |
| Medium |  | 1.350 | 1.348 |
| 200 minutes after NP injection | ***d* (µm)** | ***n1*** | ***n2*** |
| Layer #2 | 2.52 | 1.345 + 0.0030i | 1.345 + 0.0019i |
| Medium |  | 1.350 | 1.348 |
